# Trajectory of instantaneous axis of rotation in fixed lumbar spine with instrumentation

**DOI:** 10.1186/s13018-017-0677-x

**Published:** 2017-11-16

**Authors:** Masataka Inoue, Tetsutaro Mizuno, Toshihiko Sakakibara, Takaya Kato, Takamasa Yoshikawa, Tadashi Inaba, Yuichi Kasai

**Affiliations:** 1 Department of Mechanical Engineering, Graduate School of Engineering, Mie University, 1577 Kurimamachiya-cho, Tsu City, 514-8507 Mie prefecture Japan; 2 Department of Spinal Surgery and Medical Engineering, Mie University Graduate School of Medicine, 2-174 Edobashi, Tsu City, 514-8507 Mie prefecture Japan; 3 Community-University Research Cooperation Center, Mie University, 1577 Kurimamachiya-cho, Tsu City, 514-8507 Mie prefecture Japan

**Keywords:** Biomechanics, Lumbar spine, Animal experiment, Spinal instrumentation, Instantaneous axis of rotation, Trajectory

## Abstract

**Background:**

Several studies showed instantaneous axis of rotation (IAR) in the intact spine. However, there has been no report on the trajectory of the IAR of a damaged spine or that of a fixed spine with instrumentation. It is the aim of this study to investigate the trajectory of the IAR of the lumbar spine using the vertebra of deer.

**Methods:**

Functional spinal units (L5–6) from five deer were evaluated with six-axis material testing machine. As specimen models, we prepared a normal model, a damaged model, and a pedicle screw (PS) model. We measured the IAR during bending in the coronal and sagittal planes and axial rotation. In the bending test, four directions were measured: anterior, posterior, right, and left. In the rotation test, two directions were measured: right and left.

**Results:**

The IAR of the normal model during bending moved in the bending direction. The IAR of the damaged model during bending moved in the bending direction, but the magnitude of displacement was bigger compared to that of the normal model. In the PS model, the IAR during bending test hardly moved. During rotation test, the IAR of the normal model and PS model located in the spinal canal, but the IAR of the damaged model located in the posterior part of the vertebral body.

**Conclusions:**

In this study, the IAR of damaged model was scattering and that of PS model was concentrating. This suggests that higher mechanical load applied to the dura tube and nerve roots in the damaged model and less mechanical load applied to that in the PS model.

## Background

The instantaneous axis of rotation (IAR) is one of the evaluation metrics used in spinal biomechanics. Usually, the motion of a rigid body comprises translational motion and rotational motion. By regarding translational motion as rotational motion having a rotation radius of infinite length, the motion of a rigid body can be represented by the rotation around a certain point. Applying this principle to the spine, the motion of a functional spinal unit can be represented by the rotation around a point. The magnitude of the displacement of the rotating object is proportional to the horizontal distance from the axis of rotation, and the displacement is larger in positions farther from the IAR. By examining the IAR, it is possible to know the deformation behavior of the spine. Moreover, we can evaluate spinal motion characteristics in detail to investigate the trajectory of the IAR (t-IAR).

There have been numerous studies on the IAR of the lumbar spine. White et al. reported the position of the IAR during bending and rotation of an intact spine [1]. Sakamaki et al., Sengupta et al., and Haher et al. examined the IAR of the lumbar spine with damaged intervertebral disc and facet joint [2–4]. Alapan et al. investigated the effect of ligament failure on the IAR in the lower lumbar spine [5]. Orribo et al. and Huang et al. examined the IAR of a fixed spine and a spine with a replaced disc [6, 7]. Collectively, the results from these studies show that the IAR of the lumbar spine is located in stable direction.

Although the IAR seems to remain stationary during exercise load, Wachowski et al. and Mansour et al. reported that the IAR moves constantly during bending and rotation of an intact spine [8, 9]. However, there have been few reports on the t-IAR of a damaged spine or that of a fixed spine with instrumentation [10]. This study was conducted for the purpose of discussing the clinical problems of the unstable spine or the spine fixed by instrumentation by determining the t-IAR.

In this study, we used deer spine as a specimen. Since it cannot be said that the autopsy of the spine is approximate between deer and human, it is impossible to compare the biomechanics data simply by range of motion (ROM). As described by Wasinpongwanich et al., however, when the ROM change rate, an index to evaluate how the intervertebral stability will change when the normal spine of deer is injured or fixed by instrumentation, is examined, the ROM change rate in the normal, damaged, and PS fixation models in deer approximates very much to that of humans [11]. In the experiment to explore the biomechanical tendency like this study, the spine of culled deer is therefore considered available as an alternative of humans [12–14].

## Methods

**Fig. 1:**
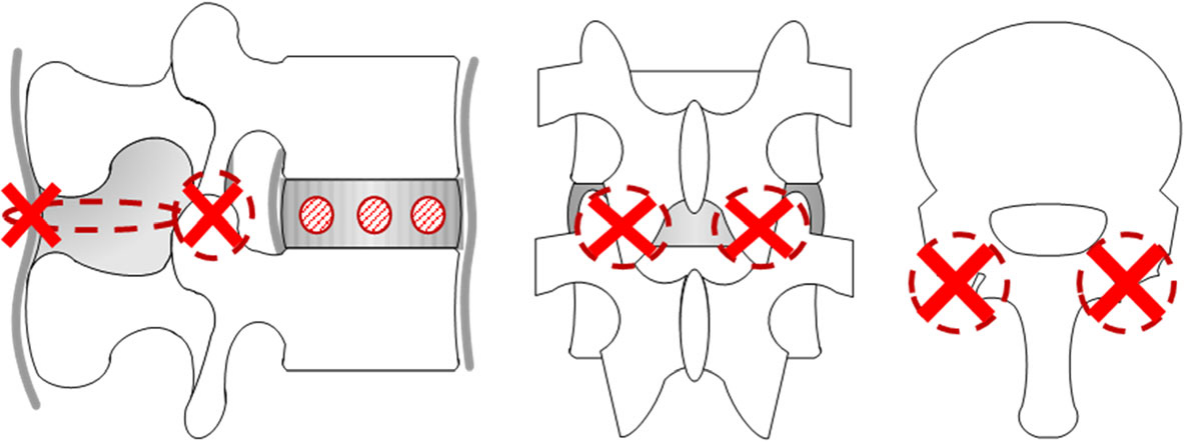
Damaged model

**Fig. 2:**
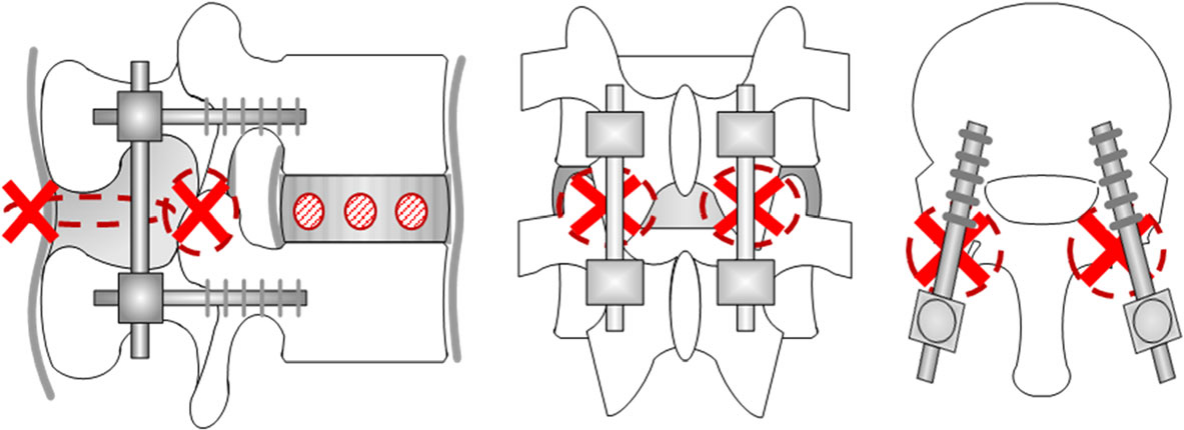
Pedicle screw model

Functional spinal units (L5–6) from five deer were used as specimens. Because L5–6 is the biggest in deer lumbar spine, damaged models or PS fixation models may be made easily. The deer were culled as part of a wildlife management program. After thawing each of the frozen lumbar spines at room temperature, the muscles and fat were removed while retaining the internal stabilizing elements. The cranial and caudal portions of each specimen were fixed to the jig with dental resin. As specimen models, we stepwisely prepared a normal model, a damaged model, and a pedicle screw (PS) model. Internal stabilizing elements were retained in the normal model. The damaged model was made by drilling through holes (diameter: 3 mm) at sites 1/4, 1/2, and 3/4 of the distance from the anterior surface on the L5/6 vertebral disc and removing its supraspinous ligament, interspinous ligament, and both facet joints (Fig. 1). The PS model was similar to the damaged model but fixed with 6.5 × 40 mm PSs and rods (KiSCO: S-LineII, Saint-Priest, France) (Fig. 2).

**Fig. 3:**
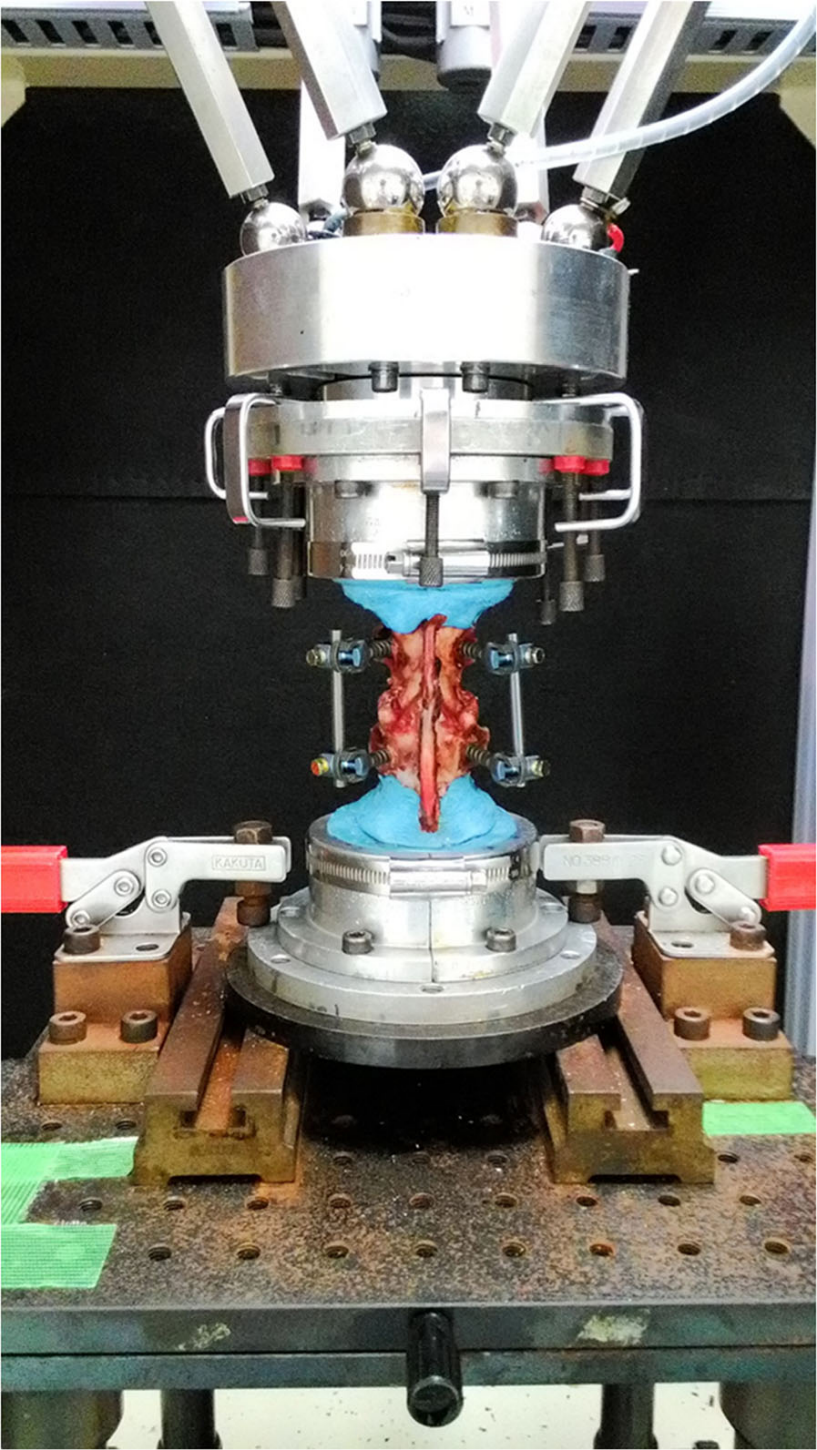
Six-axis material testing machine

For the tests, a six-axis material testing machine developed in our laboratory was used (Fig. 3) [15]. This testing machine adopts a parallel mechanism. A set of two actuators is located parallel at 120° to the object, and each of the six actuators is independently controlled. At the lower end of six actuators, a six-axis kinesthetic sensor is equipped to detect forces in the *x*-, *y*-, and *z*-axes and the torque around each axis. Furthermore, this kinesthetic sensor enables force control by feeding back the detected values to the control system and enables motion with multiple degrees of freedom.

Bending in the coronal and sagittal planes (bending test) and axial rotation (rotation test) were conducted for each model using this testing machine. In the bending test, linear and angular displacements were measured: anterior, posterior, right, and left. In the rotation test, two directions were measured: right and left. The torque was set at 3.0 Nm for the bending test and 4.0 Nm for the rotation test. Each test was repeated twice. And 100 N axial preloads are provided in all tests. The number of degrees of freedom in the bending test was set to three to allow genuine bending in one plane. The number of degrees of freedom in the rotation test was set to four to allow displacement along the *x*-, *y*-, and *z*-axes and rotation around the *z*-axis.

**Fig. 4:**
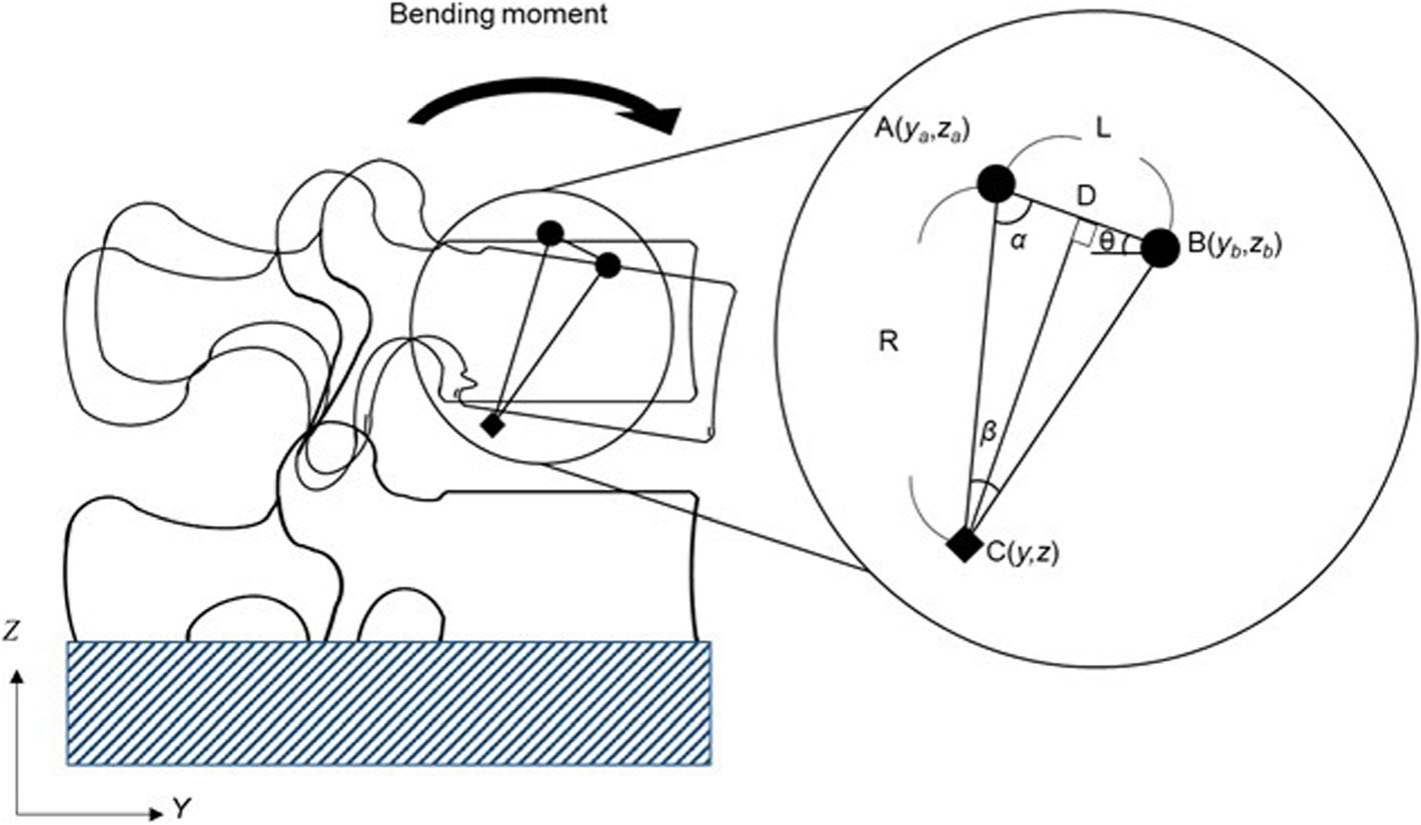
Determination of instantaneous axis of rotation

Linear and angular displacements from the time of no load to the time of maximum torque during the bending and rotation tests were measured. The IAR was calculated for every 0.2-degree increment of angular displacement. To calculate the IAR, the angular displacement and position coordinates for before and after motion in a corresponding section were used. An example of calculating the IAR (point C) when the position coordinates change from point A to point B is shown in Fig. 4. The position coordinates of point A, point B, and the angle *β* are obtained from the testing machine. First, the length *L* and the angle *θ* formed by the line segment AB and the horizontal plane are determined using Eqs. (1) and (2).

1 $$ L=\sqrt{{\left({y}_b-{y}_a\right)}^2+{\left({z}_b-{z}_a\right)}^2} $$

2 $$ \theta ={\tan}^{-1}\left({y}_b-{y}_a/{z}_b-{z}_a\right) $$

Next, consider the triangle ACD comprising point A, point C shown in Fig. 4, the line segment AB, and point D, which is a point of intersection of the line segment AB and its vertical bisector. The angle *α* is obtained from the sum of the interior angles of the triangle. The length *R* is calculated from the trigonometric ratio (Eq. (3)).

3 $$ R=\frac{L}{2\sin \frac{\beta }{2}} $$

The position coordinates of point C are obtained from Eq. (4). Point C is distance *R* away from point A. The angle of point C is *θ* + *α* to the *x*-axis.

$$ y={y}_a+R\cos \left(\theta +\alpha \right) $$

4 $$ z={z}_a-R\kern.15em \sin \left(\theta +\alpha \right) $$

We calculate the IAR from data of second reciprocating motion of the bending and rotation. In the anterior–posterior bending test, *β* represents the angular displacement around the *x*-axis of the upper vertebral body compared to the angular displacement of the lower vertebral body, and (*y*_*b*_ − *y*_*a*_) and (*z*_*b*_ − *z*_*a*_) represent the magnitude of translation in the *y*- and *z*-axis directions of the upper vertebral body compared to the magnitude of translation of the lower vertebral body, respectively. The IAR during the bending and rotation tests can also be calculated using the magnitudes of angular displacement and translation from the upper vertebral body compared to the magnitudes of angular displacement and translation of the lower vertebral body.

**Fig. 5:**
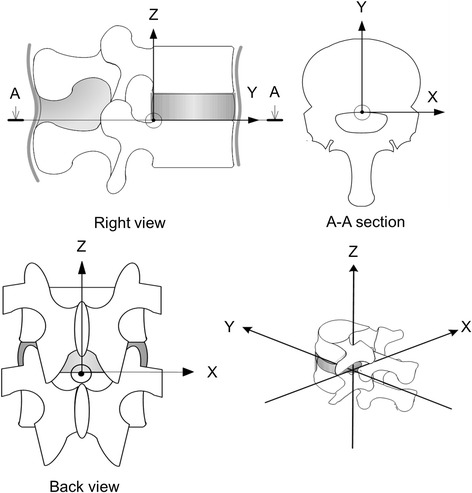
The axis of coordinates

**Fig. 6:**
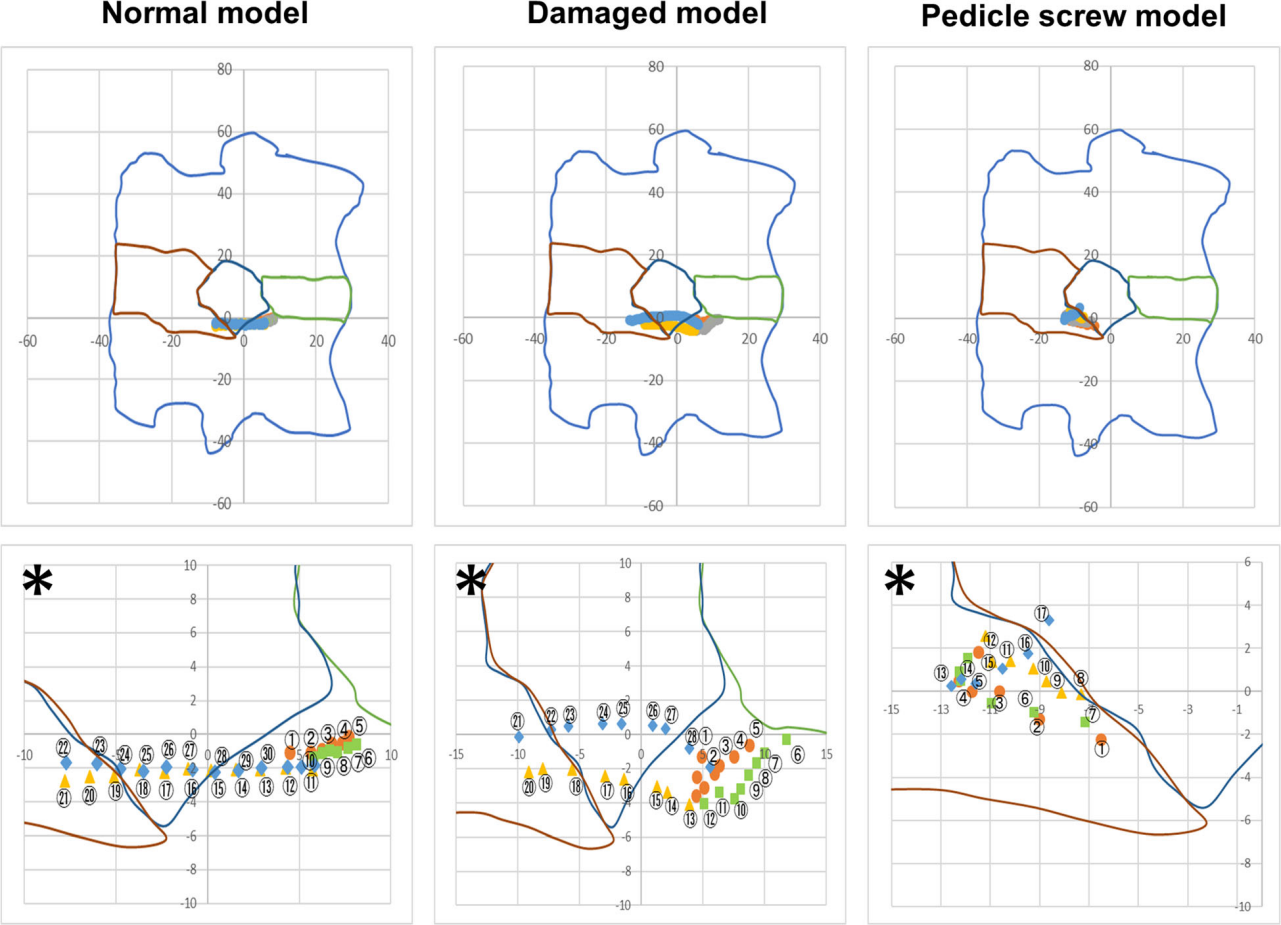
Trajectory of IAR during anterior–posterior bending. Asterisk shows enlarged picture of above

**Fig. 7:**
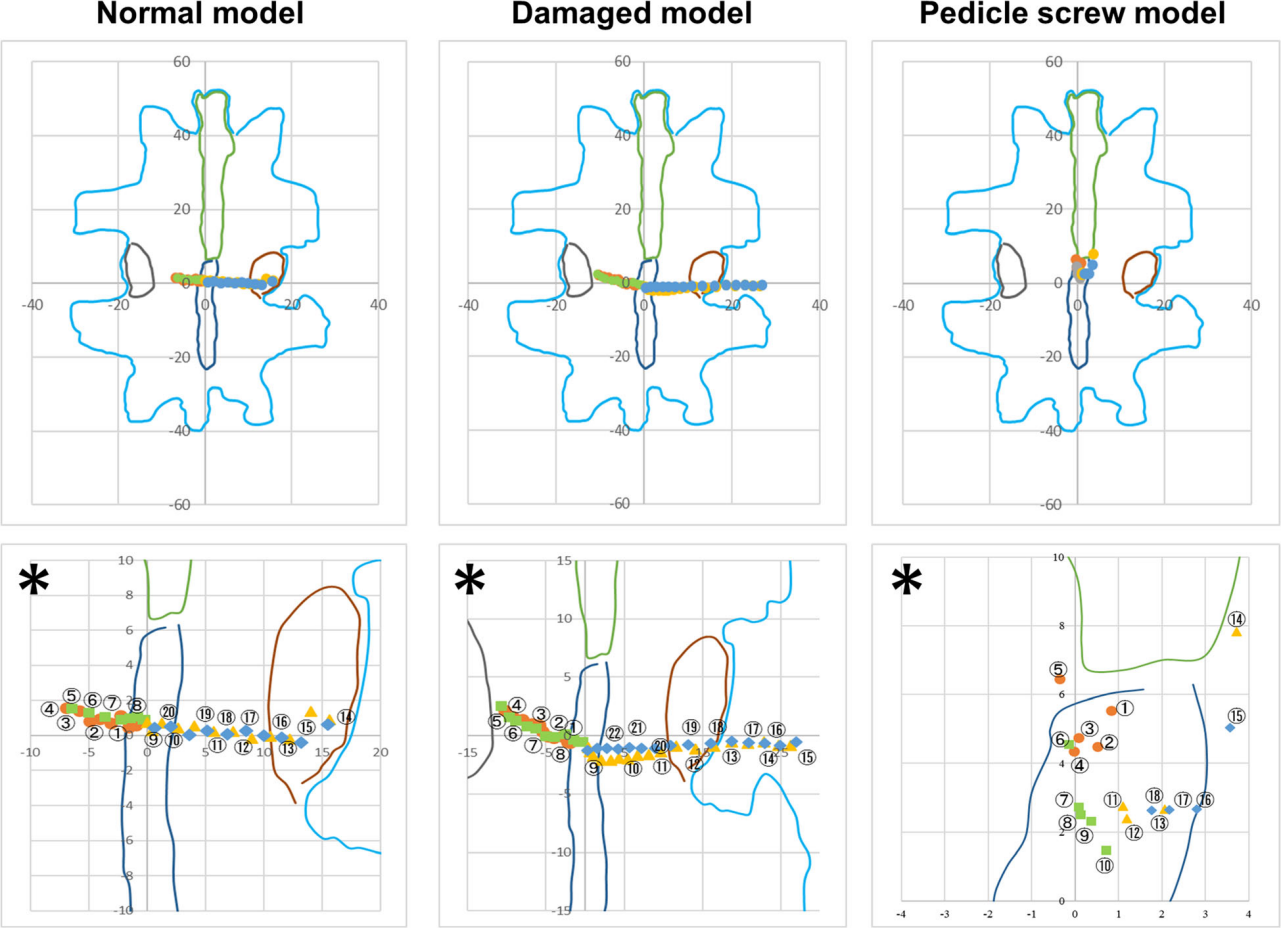
Trajectory of IAR during lateral bending. Asterisk shows enlarged picture of above

**Fig. 8:**
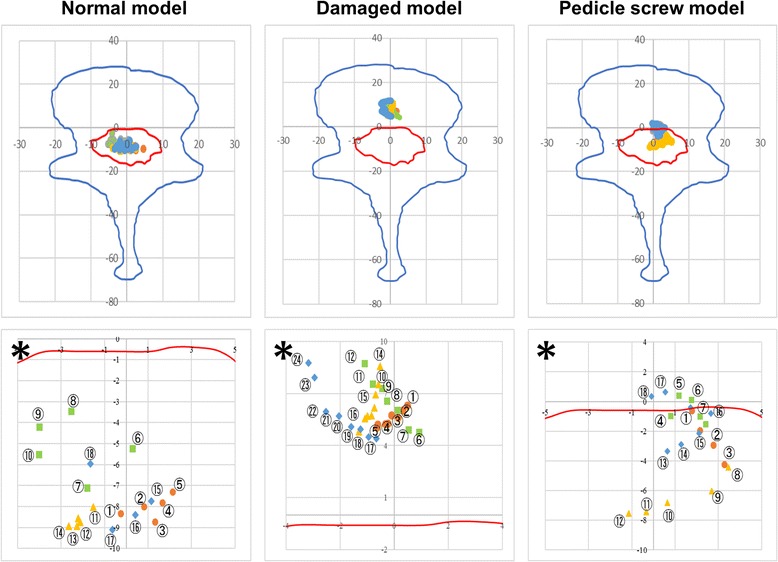
Trajectory of IAR during rotation. Asterisk shows enlarged picture of above

The t-IAR in the anterior–posterior bending test was overlaid on the coordinate system, in which the caudal posterior end of the intervertebral disc is the origin *O*, the anterior–posterior direction of the vertebra is the *y*-axis (anterior is positive), and the cranial-to-caudal side direction is the *z*-axis (cranial side is positive). The t-IAR in the left–right bending test was overlaid on the coordinate system, in which the midpoint of the left and right horizontal diameters of the intervertebral disc on the caudal posterior edge is the origin *O*, the left–right direction of the vertebra is the *x*-axis (right side is positive), and the cranial-to-caudal direction is the *z*-axis (cranial side is positive). Further, the t-IAR in the rotation test was overlaid on the coordinate system, in which the midpoint of the left and right horizontal diameters of the vertebral body on the posterior edge is the origin *O*, the left–right direction of the spine is the *x*-axis (right side is positive), and the anterior–posterior direction is the *y*-axis (anterior is positive) (Fig. 5). The means of the t-IAR of five specimens are plotted in Figs. 6, 7, and 8.

## Results

### Anterior–posterior bending

Figure 6 shows the IAR during anterior–posterior bending. In this figure, the trace plotted with circles represents the t-IAR during anterior bending, the trace plotted with squares represents the t-IAR when returning to the midline after anterior bending, the trace plotted with triangles represents the t-IAR during posterior bending, and the trace plotted with rhomboids represents the t-IAR when returning to midline after posterior bending. The numbers in Fig. 1 indicate the order of movement of the t-IAR. Each of the five specimens tended to exhibit the same shift of the t-IAR during anterior–posterior bending.

The IAR of the normal and damaged models tends to be located in the anterior region of the vertebral body during anterior bending and in the posterior region of the vertebral body during posterior bending. On the other hand, the IAR of the PS model is in the posterior region of the spine. Particularly, the IAR during posterior bending is in a cranial position compared with the IAR during anterior bending. The t-IAR of the damaged model during anterior–posterior bending is longer than that of the normal model. On the other hand, the t-IAR of the PS model is shorter than that of the normal and damaged models.

### Left–right bending

Figure 7 shows the IAR during left–right bending. In this figure, the trace plotted with circles represents the t-IAR during bending to the left, the trace plotted with squares represents the t-IAR when returning to midline after bending to the left, the trace plotted with triangles represents the t-IAR during bending to the right, and the trace plotted with rhomboids represents the t-IAR when returning to midline after bending to the right. The numbers in Fig. 2 indicate the order of movement of the t-IAR. Each of the five specimens tended to exhibit the same shift of the t-IAR during left–right bending.

The IAR of the normal and damaged models during left–right bending is located on the left side of the vertebral body during bending to the left and on the right side of the vertebral body during bending to the right. On the other hand, the IAR of the PS model is primarily located in the center of the vertebral body. While the t-IAR of the normal and damaged models during left–right bending is in the intervertebral disc, the t-IAR of the PS model is in a cranial position. The t-IAR of the damaged model is longer than that of the normal model. In contrast, the t-IAR of the PS model is shorter than that of the normal and damaged models.

### Rotation

Figure 8 shows the IAR during rotation. In this figure, the trace plotted with circles represents the t-IAR during rotation to the left, the trace plotted with squares represents the t-IAR when returning to midline after rotation to the left, the trace plotted with triangles represents the t-IAR during rotation to the right, and the trace plotted with rhomboids represents the t-IAR when returning to midline after rotation to the right. Each of the five specimens tended to exhibit the same shift of the t-IAR during rotation.

Now, t-IAR always exists in the spinal canal in the normal model and PS model in axial rotation, but it transfers anteriorly into the vertebral body in the damaged model. In the damaged model, moreover, t-IAR does not move so much in comparison with the other models. From the above, it is considered that the normal model and PS model have a small dynamic load onto the dural tube in the spinal canal, but in the damaged model, a load is always put on the dural tube. It is therefore presumed that persistent dynamic stress is placed on the dural tube when intervertebral instability is observed.

## Discussion

This study is the first to examine the t-IAR of a damaged lumbar spine and instrumented spine during bending in the coronal and sagittal planes and axial rotation.

According to the results of the present study, the IAR of the normal model during bending moves in the bending direction, but remains in the spinal canal during rotation. These results agree with that from a study by Wachowski et al., who studied the kinematics of spinal segment [8]. Further, since the t-IAR of the normal model during bending and rotation remains in the spinal canal, the displacement and shear load occurring in the dura mater tube and nerve roots in the spinal canal are considered small.

Similar to the normal model, the IAR of the damaged model during bending moved in the bending direction. However, the magnitude of displacement of the IAR of the damaged model is bigger compared to that of the normal model, and the IAR is away from the spinal canal. Thus, the shear load occurring in the dura mater tube and nerve roots of the damaged model is higher than that of the normal model. Ahmadi et al. also reported that arc length of instantaneous center of rotation was significantly higher in patients with low back pain, and this might be one of the causes of low back pain or nervous symptoms [16]. The t-IAR during rotation is primarily located in the vertebral body. This suggests that the rigidity in the posterior region of the spine is decreased because of the damage to both facet joints, and the rigidity in the anterior region of the spine is relatively increased. The IAR was primarily located in the spinal canal in the normal model, but shifted to the vertebral body in the damaged model. Higher shear load is applied to the dura mater tube or nerve roots in the spinal canal and may worsen neurological symptoms. Therefore, IAR analysis reconfirms that fusion surgery is necessary for trauma with facet joint injury or patients with degenerative disease.

In the PS model, the IAR during anterior–posterior bending is primarily located in the posterior region of the spine. This is likely caused by the PS instrumentation increasing the rigidity in this region. During anterior–posterior bending of the spine, a high load might have been applied to the front of the vertebra and intervertebral disc, as well as the anterior tip of the PS. Further, the t-IAR during posterior bending shifts to a cranial position compared with the t-IAR during anterior bending. This suggests that a high load might have been applied to the cranial region of the specimen during anterior bending and to the caudal region of the specimen during posterior bending of the spine instrumented with PS. During left–right bending, the IAR is primarily located in the center of the vertebral body in the PS model, which seems to be the ideal position. Further, during rotation, the IAR is primarily located in the posterior region of the vertebral body in the vicinity of the spinal canal, and instrumentation with PS is considered to reduce the mechanical load applied to the dura tube or nerve roots.

This study has several limitations: (1) the specimens were spinal columns from deer, (2) only five samples were tested, (3) PSs for humans were used, and (4) coupling motion was not considered. In the future, we plan to conduct similar experiments using human cadavers, increase the number of samples, and perform experiments and repeated loading tests for the coupling motion.

## Conclusion

We examined the t-IAR in different spine models subjected to bending and rotation. The model with damage to the intervertebral disc and facet joint exhibited increased intervertebral instability, which led to higher mechanical load on the dura tube or nerve roots. The mechanical load on the dura tube or nerve roots was reduced in the model with PS instrumentation, but this model exhibited a higher mechanical load on the front of the vertebral body and intervertebral disc and on the anterior tip of the PS during anterior–posterior bending.

## Abbreviations

IAR: Instantaneous axis of rotation
PS: Pedicle screw
ROM: Range of motion
t-IAR: Trajectory of the IAR
